# Supplementary material for: Fast-diffusing receptor collisions with slow-diffusing peptide ligand assemble the ternary parathyroid hormone–GPCR–arrestin complex
Source: Nat Commun. 2024 Dec 3;15:10499. doi: 10.1038/s41467-024-54772-3 (PMC11615292; doi:10.1038/s41467-024-54772-3)
Supplement: Supplementary file 1 — Supplementary Information [file 41467_2024_54772_MOESM1_ESM.pdf]

Supplementary Information for

Fast-diffusing receptor collision with slow-diffusing peptide ligand assemble the ternary parathyroid hormone–GPCR–arrestin complex

Jonathan Pacheco[1], Karina A. Peña[1], Sofya Savransky[1,2], Alexandre Gidon[1‡], Gerald R.V. Hammond[3], John Janetzko[5] & Jean-Pierre Vilardaga[1,2,4*]

[1]Department of Pharmacology and Chemical Biology, [2]Graduate Program in Molecular Pharmacology, and [3]Department of Cell Biology, University of Pittsburgh School of Medicine, Pittsburgh PA 15261, USA. [4]U.S. Department of Veterans Affairs, Pittsburgh Healthcare System, Pittsburgh, PA 15240, USA. [5]Department of Molecular and Cellular Physiology, Stanford University School of Medicine, Stanford, CA 94305, USA

*Jean-Pierre Vilardaga
Email: jpv@pitt.edu

This PDF file includes:

Supplementary Tables 1 to 3
Supplementary Figures 1 to 14

Supplementary Table 1. Fluorescent-labeled peptides used in this study.

	1 5 10 15 20 25 30
**Peptides**	**sequences**
PTH^{TMR}	SVS EIQLMHNL G K^{tmr}HLNSMERVEWLRKKLQDVHNF
IA-PTH^{TMR} (L$_{11}$,dW$_{12}$,W$_{23}$,Y$_{36}$-PTHrP$_{7\text{-}36}$)	LLHDL W^{d}K^{tmr}SIQDLRRRFWLHHLIAEIHTAEY
LA-PTH^{TMR}	AVA EIQLMHQR A K^{tmr}WIQDARRRAFLHKLIAEIHTAEI
M-PTH$_{1\text{-}14}$^{TMR}	AVA*EIQLMHQH*A K^{tmr}W
PTH^{Fluo}	SVS EIQLMHNL G K^{fluo}HLNSMERVEWLRKKLQDVHNF

A*: Aib, 2-Aminoisobutyric acid
H*: Har, homoarginine
TMR: tretramethylrhodamine
Fluo: fluorescein

Supplementary Table 2. Plasmids used in this study.

Plasmid	Backbone	Insert	References
mNGx1-PH-PLCδ1	pmNG2-N1(Truncated CMV promotor)	PLCD1v2(1-170):mNG2	Wills et. al. 2022
mNGx2-PH-PLCδ1	pmNG2-N1(Truncated CMV promotor)	PLCD1v2(1-170):mNG2:mNG2	Wills et. al. 2022
mNGx3-PH-PLCδ1	pmNG2-N1(Truncated CMV promotor)	PLCD1v2(1-170):mNG2:mNG2:mNG2	Wills et. al. 2022
mNG-β-arrestin2	pmNG2-N1(Truncated CMV promotor)	ARRB2:GGVGG:mNG2	This study
mNG-N1-PTH$_1$R	pmNG2-N1	PTH1R:GGGGGG:mNG2	This study
NES-mCherry-PH-ARNO$^{2G\text{-}I303E}$x2	pNES-mCherry-C1	X. laevis map2k1.L(32-44):mCherry:CYTH2(252-399,I303E):GGSGGVDM:CYTH2(252-399,I303E)	Goulden et. al., 2018
pECFP-N1-PTH$_1$R	pECFP-N1	PTH1R:GGGGGG:CFP	Peña. et. al. 2022
pEYFP-β-arrestin2	pEYFP-N1	ARRB2:TCTAGA:YFP	Vilardaga et. al. 2002
PAmCherry1-β-arrestin2	pPAmCherry-N1(Truncated CMV promotor)	ARRB2:GGVGG:PAmCherry1	This study
iRFP670-N1-PTH$_1$R	iRFP670-N1	PTH1R:GGGGGG:iRFP670	This study
iRFP670-N1-PTH$_1$R^{PD}	iRFP670-N1	PTH1R(S489A,S491A,S492A,S493A,S495A):GGGGGG:iRFP670	This study

Supplementary Table 3. Diffusion coefficients, motion, and calculated time to diffuse to CCPs for the model in Fig. 9

	Ligand	Diffusion coefficients ($10^{-3}\mu m^2/s$)	Motion (α)	Area ($\mu m^2/min$)
**PTH_1R^{mNG}**	none	88.64 ± 11	0.72 ± 0.03	**6.8**
**PTH_1R^{iRFP}**	none	64.06 ± 6.5	0.61 ± 0.027	**3.2**
**$\beta arr2^{mNG}$**	none	16.36 ± 3.0	0.44 ± 0.02	**0.4**
**PTH_1R^{mNG}**	PTH^{TMR}	2.7 ± 0.6	0.23 ± 0.05	0.029
**PTH_1R^{iRFP}**	PTH	5.2 ± 0.6	0.27 ± 0.02	0.065
**$\beta arr2^{mNG}$**	PTH^{TMR}	3.1 ± 0.4	0.32 ± 0.03	0.048
**PTH^{TMR} (PTH_1R^{mNG} cells)**	n/a	1.8 ± 0.1	0.28 ± 0.04	0.024

mean ± SEM. n/a, not applicable. In bold letter, the area greater than the average empty circles of clathrin (0.38 ± 0.016 µm2)

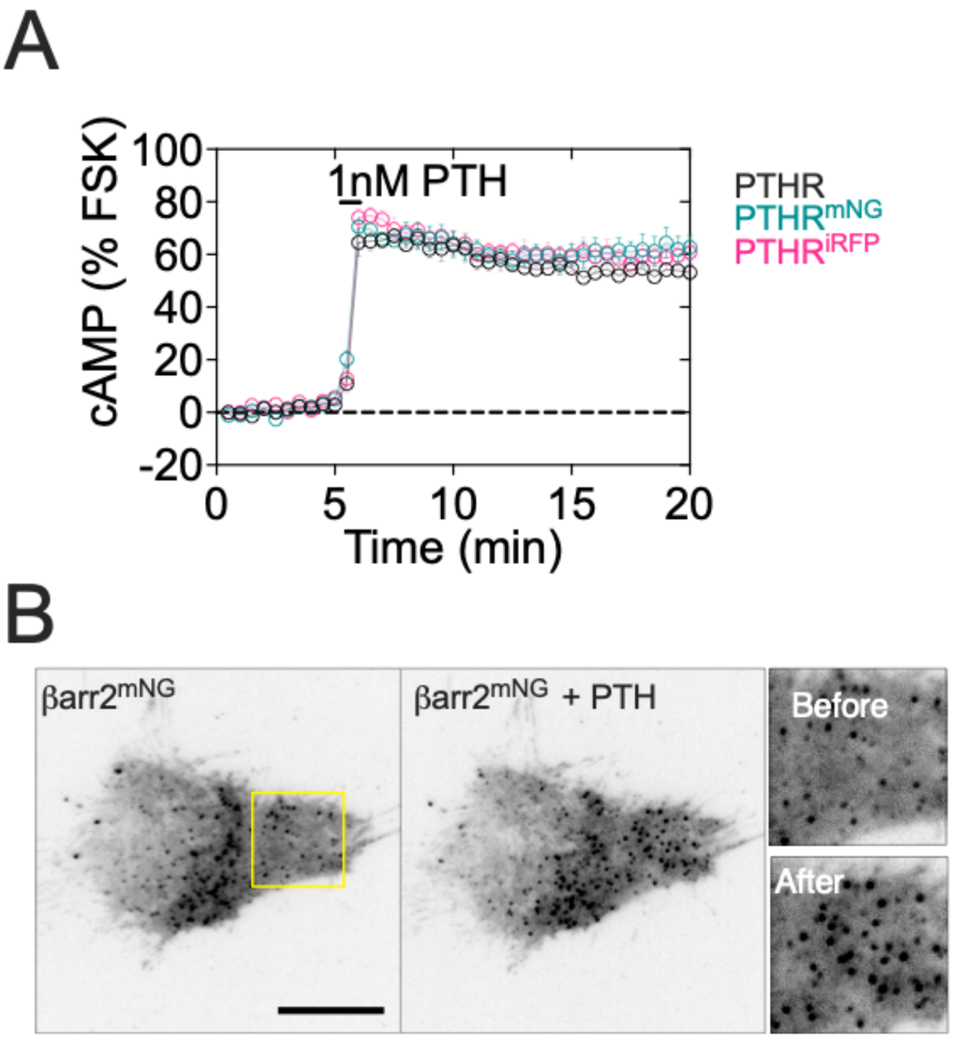

Supplementary Fig. 1| Validation of constructs.

(A) Time courses of cAMP in single HEK-293 cells. Cells expressing the fluorescent constructs PTH$_1$R^{PAmCherry}, PTH$_1$R^{iRFP}, or PTH$_1$R^{mNG} used for single-molecule imaging. Cells were briefly perfused with 1 nM PTH (horizontal bar). The percentage of cAMP responses is relative to the response in the presence of 5 μM forskolin (FSK). Data are means ± SD of N = 3 experiments with n = 10 cells/experiments.

(B) Recruitment of βarr2^{mNG} to the plasma membrane of HEK293 cells expressing PTH$_1$R^{iRFP} and βarr2^{mNG}. Cells were imaged by TIRF microscopy before and after the addition of PTH 10 nM. The detail (right) corresponds to the yellow region of interest (ROI), which shows the increase in the number of βarr2^{mNG} spots. Scale bar: 10 μm.

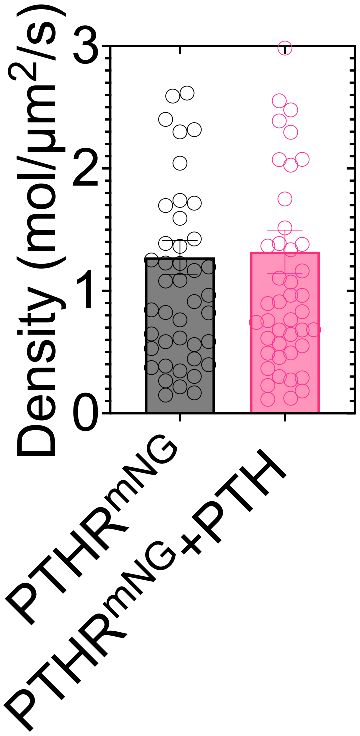

Supplementary Fig. 2| Average density of molecules in experiments of single molecule. The average number of molecules was normalized by the area of cell per second. Data are the mean ± SEM of *n* = 46 (PTH_1R^{mNG}) and *n* = 47 (PTH_1R^{mNG} + PTH) cells from N = 6 independent experiments.

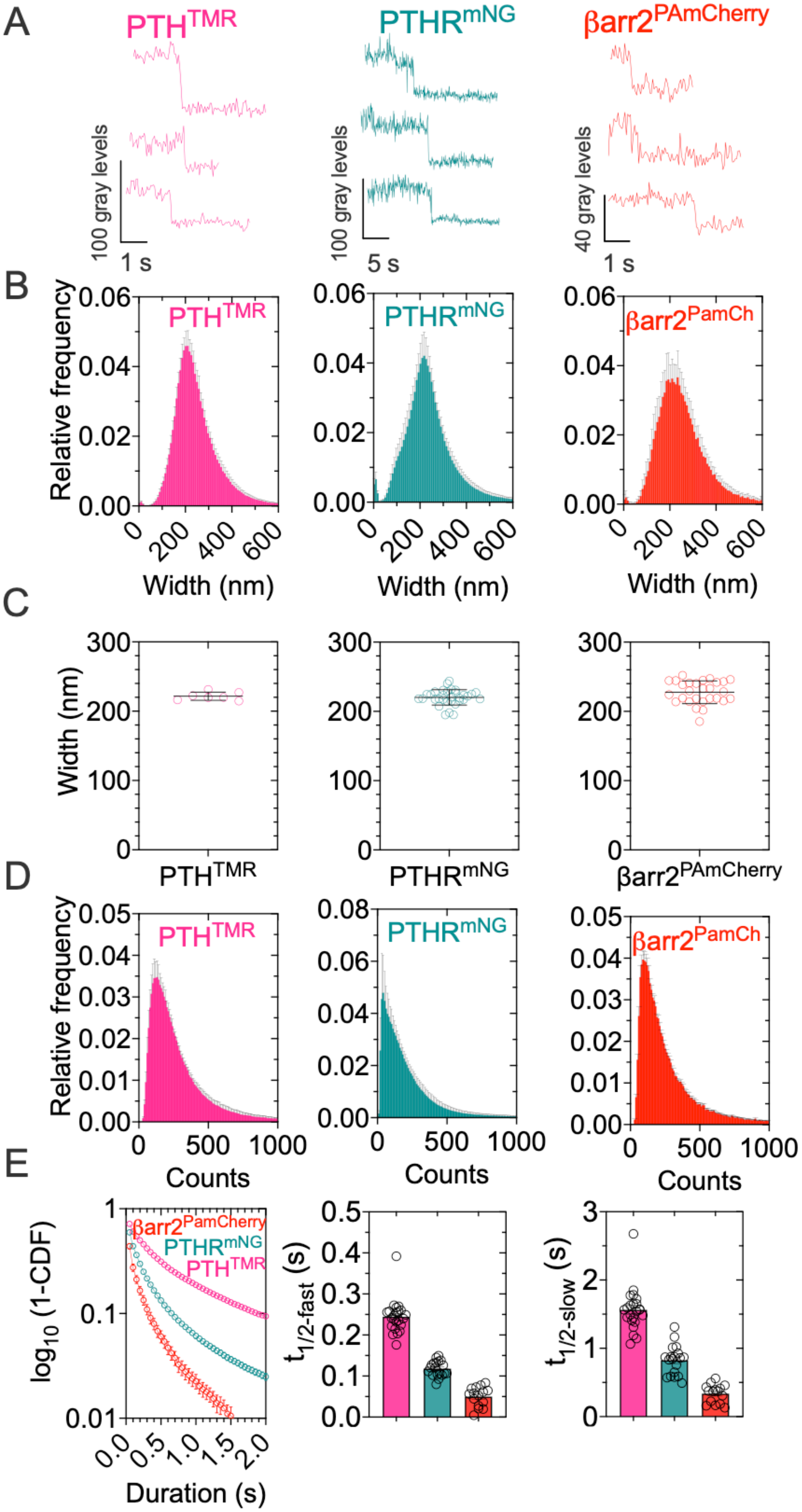
A
PTH^TMR
PTHR^mNG
βarr2^PAmCherry
100 gray levels
1 s
100 gray levels
5 s
40 gray levels
1 s
B
Relative frequency
0.06
0.04
0.02
0.00
0
200
400
600
Width (nm)
PTH^TMR
PTHR^mNG
βarr2^PamCh
C
Width (nm)
300
200
100
0
PTH^TMR
PTHR^mNG
βarr2^PAmCherry
D
Relative frequency
0.05
0.04
0.03
0.02
0.01
0.00
0.08
0.06
500
1000
Counts
E
log10 (1-CDF)
1
0.1
0.01
0.0
0.5
1.0
1.5
2.0
Duration (s)
t1/2-fast (s)
0.5
0.4
0.3
0.2
0.1
t1/2-slow (s)
3
2
1

Supplementary Fig. 3 | Validation of single-molecule recordings.
(A) Representative fluorescence profiles showing a single-step photobleaching for PTH^{TMR} PTH_1R^{mNG} and $\beta arr2^{PAmCherry}$.
(B, C) Diffraction-limited size of individual fluorescent spots for PTH^{TMR}, PTH_1R^{mNG} and $\beta arr2^{PAmCherry}$. The distribution of the diameter of spots per cell was fit using a Gaussian function (B) to calculate the average size of particles (C).
(D) Distribution of brightness of individual fluorescent spots for PTH^{TMR}, PTH_1R^{mNG} and $\beta arr2^{mNG}$. Data are mean ± S.E.M. of *n* = 32/36700 (PTH_1R^{mNG}), *n* = 7/219876 (PTH^{TMR}) and *n* = 28/86247 ($\beta arr2^{PAmCherry}$) cells/ average detections per cell from at least 3 independent experiments.
(E) Lifetime of fluorophores. Cells expressing the corresponding construct were fixed and the lifetime of the fluorophore was measured by a survival function (1-CDF). The curves were fitted with a bi-exponential model to extract the fast and slow half-lifetime. Data are mean ± SEM of *n* = 19 (PTH_1R^{mNG}), *n* = 14 (PTH^{TMR}) and *n* = 15 ($\beta arr2^{PAmCherry}$) cells.

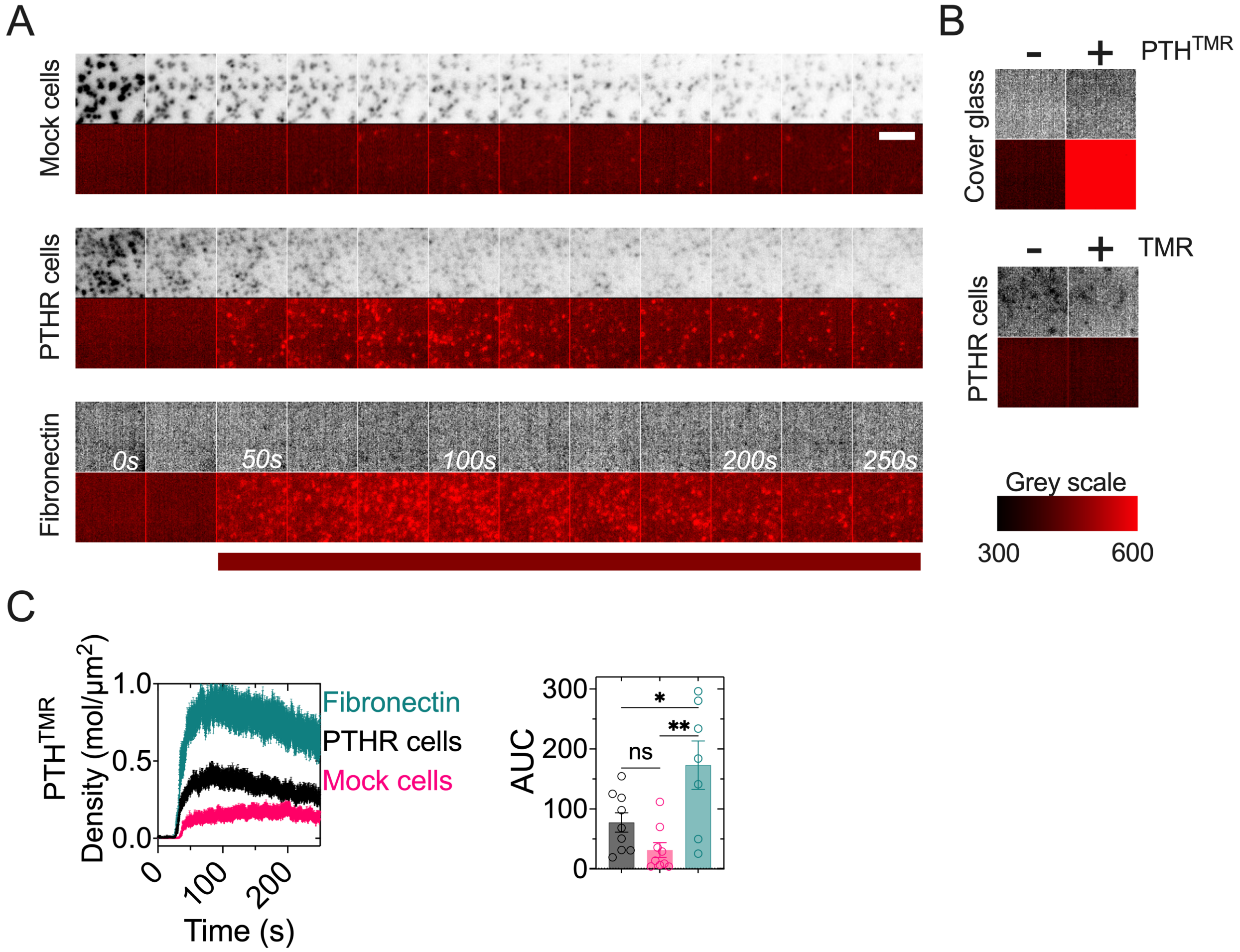

Supplementary Fig. 4| PTH^{TMR} binding kinetics.

(**A**) Representative time-lapses of PTH^{TMR} incorporation in CLTA^{mNG} cells without (Mock cells) and with expression of PTH$_1$R^{mNG} (PTH$_1$R cells), and background level in fibronectin-coated glass coverslips with no cells (Fibronectin). The horizontal red bar indicates the time point at which PTH^{TMR} was added. The mNG channel (in grayscale) was used to ensure the TIRF focus plane. Scale bar: 5 μm

(**B**) Examples showing fluorescence levels before and after adding PTH^{TMR} or TMR in uncoated glass coverslips and PTH$_1$R^{mNG} cells, respectively.

(**C**) Density time-lapses in conditions of panel A (left). The area under the curve (AUC) from time-lapses of molecule density (right). Data are mean ± S.E.M. of n = 9 (Mock), n = 9 (PTH$_1$R^{mNG}) and n = 7 (Fibronectin cover glass) cells. $^{**}P < 0.01$, $^{*}P < 0.05$ by one-way ANOVA with a Tukey's multiple comparison.

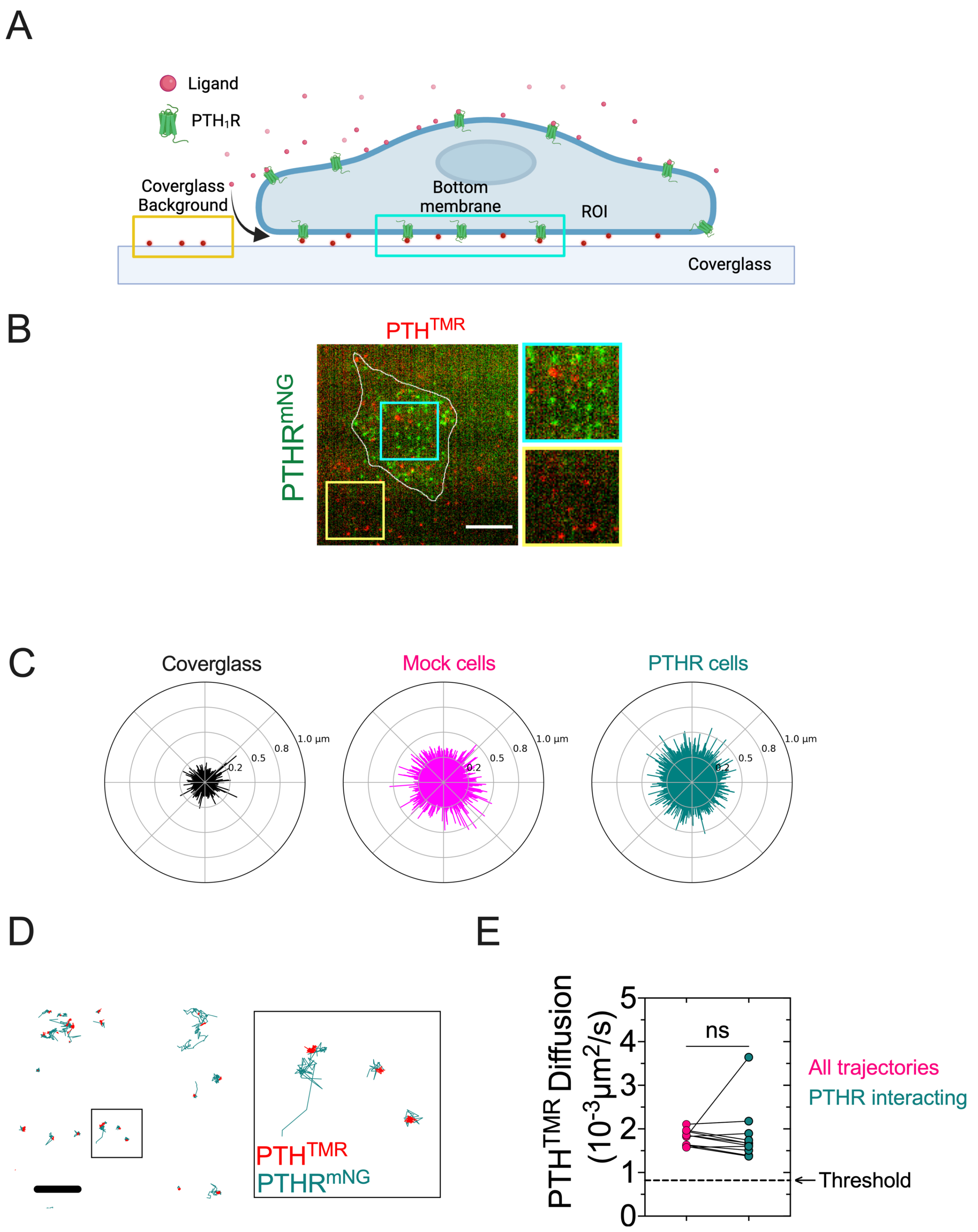
A
Ligand
PTH1R
Coverglass
Background
Bottom
membrane
ROI
Coverglass
B
PTHTMR
PTHRmNG
C
Coverglass
Mock cells
PTHR cells
1.0 µm
0.8
0.5
0.2
D
PTHTMR
PTHRmNG
E
PTHTMR Diffusion
(10-3µm2/s)
ns
All trajectories
PTHR interacting
Threshold
0
1
2
3
4
5

Supplementary Fig. 5| Single-molecule spatial, diffusion-based, and interacting-based filtering of fluorescent ligand.
(**A, B**). Scheme illustrating the spatial filtering in our approach to detect single PTH^{TMR} molecule. Regions of interest (ROI) are drawn in areas that correspond to the cell (*cyan*) and the glass coverslip (*yellow*) (A). An example of a cell expressing the PTH_1R^{mNG} stimulated with PTH^{TMR} with ROI inside (*cyan*) and outside (*yellow*) the cell. The outlined white line indicates the cell footprint generated from accumulated detections of PTH_1R^{mNG}. Scale bar: 5 µm. Scheme created in BioRender. Pacheco Romero, J. (2025) https://BioRender.com/c32f451
(**C**) The diffusion-based filter consists of the differences in the diffusion of PTH^{TMR} when it is on the coverslip or in the cell. Here, circular plots represent the total displacements of trajectories (µm) in individual cells. Data are 2248 (cover glass), 36735 (Mock cells), and 54440 (PTH_1R cells) trajectories.
(**D, E**) Interacting-based filter consists in capturing the dynamic features of only the trajectories that showed coincidental detection with the PTH_1R^{mNG}. This permits us to ensure that the ligand molecules were functional to associate with the receptor (D). Scale bar and inset: 2 µm.
Comparison of diffusion coefficient values of all trajectories of PTH^{TMR} filtered by spatial location and diffusion and the trajectories that showed coincidental detection with the receptor. The absence of differences supports the robustness of the analysis (E). *n* = 11/5751 (All trajectories), n = 11/ 777 (PTHR interacting) cells/average trajectories per cell from 4 independent days of experiment. The data presented in condition: all trajectories is the same as those shown in the panels of figures 2C and 2D ns, not significant by paired t-test.

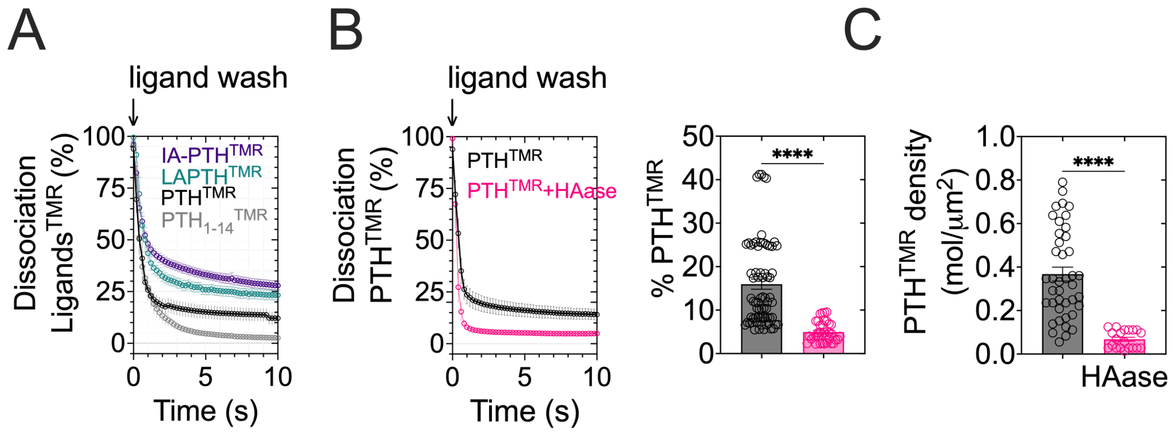

Supplementary Fig. 6| Hyaluronic acid-dependent PTH binding to the cell surface.

(A). Dissociation time courses of fluorescent ligands in non-transfected HEK-293 cells. Single cells were perfused with 1 μM of fluorescent ligand and then washed out. Data are the mean ± SEM of *n* = 10 (PTH^{TMR}), *n* = 13 ($PTH_{1\text{-}14}{}^{TMR}$), *n* = 10 ($LAPTH^{TMR}$), and *n* = 10 ($IA\text{-}PTH^{TMR}$).

(**B**) Dissociation time courses of PTH^{TMR} in non-transfected control (black) and hyaluronidase-treated (HAase, 20 μ/mL for 30 min) cells (*left*). The bar graph represents the percentage of PTH^{TMR}-bound to cells after 10 seconds of ligand washout (*right*). Data are the mean ± SEM of *n* = 17 (PTH^{TMR}), and *n* = 9 (PTH^{TMR} + HAase). ****$P < 0.0001$ by an unpaired t-test.

(**C**) Density of single molecules after 250 sec of stimulation of PTH^{TMR} in cells expressing the PTH_1R^{mNG} with or without HAase treatment. Data are the mean ± SEM. n = 41 (PTH^{TMR}), and n = 20 cells (HAase). ****$P < 0.0001$ by an unpaired t-test.

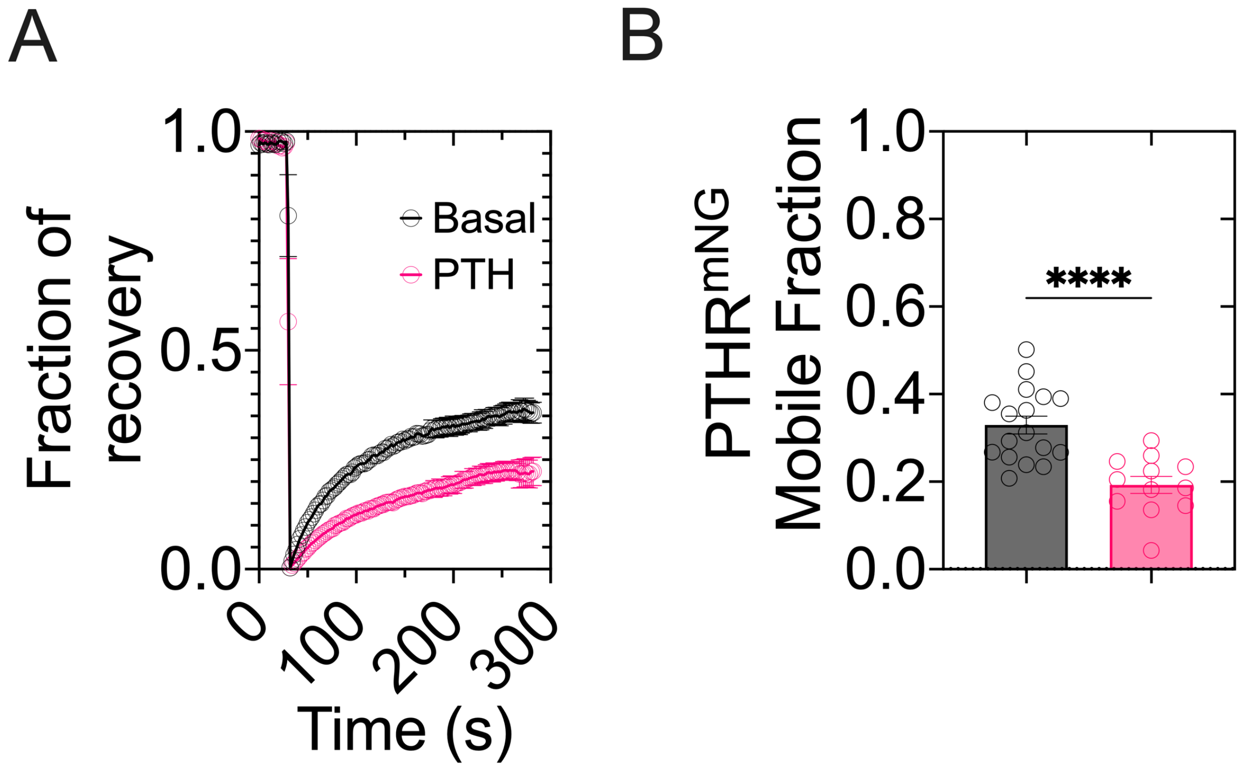

Supplementary Fig. 7| Fluorescence recovery after photobleaching of PTH$_1$R^{mNG}. (**A, B**) Time courses of fluorescence recovery (A) and quantification of mobile fraction from FRAP experiments at 200 s (A). Data are the mean ± SEM of *n* = 17 (Basal) and *n* = 13 (PTH^{TMR}) cells. ****$P < 0.0001$ by an unpaired t-test.

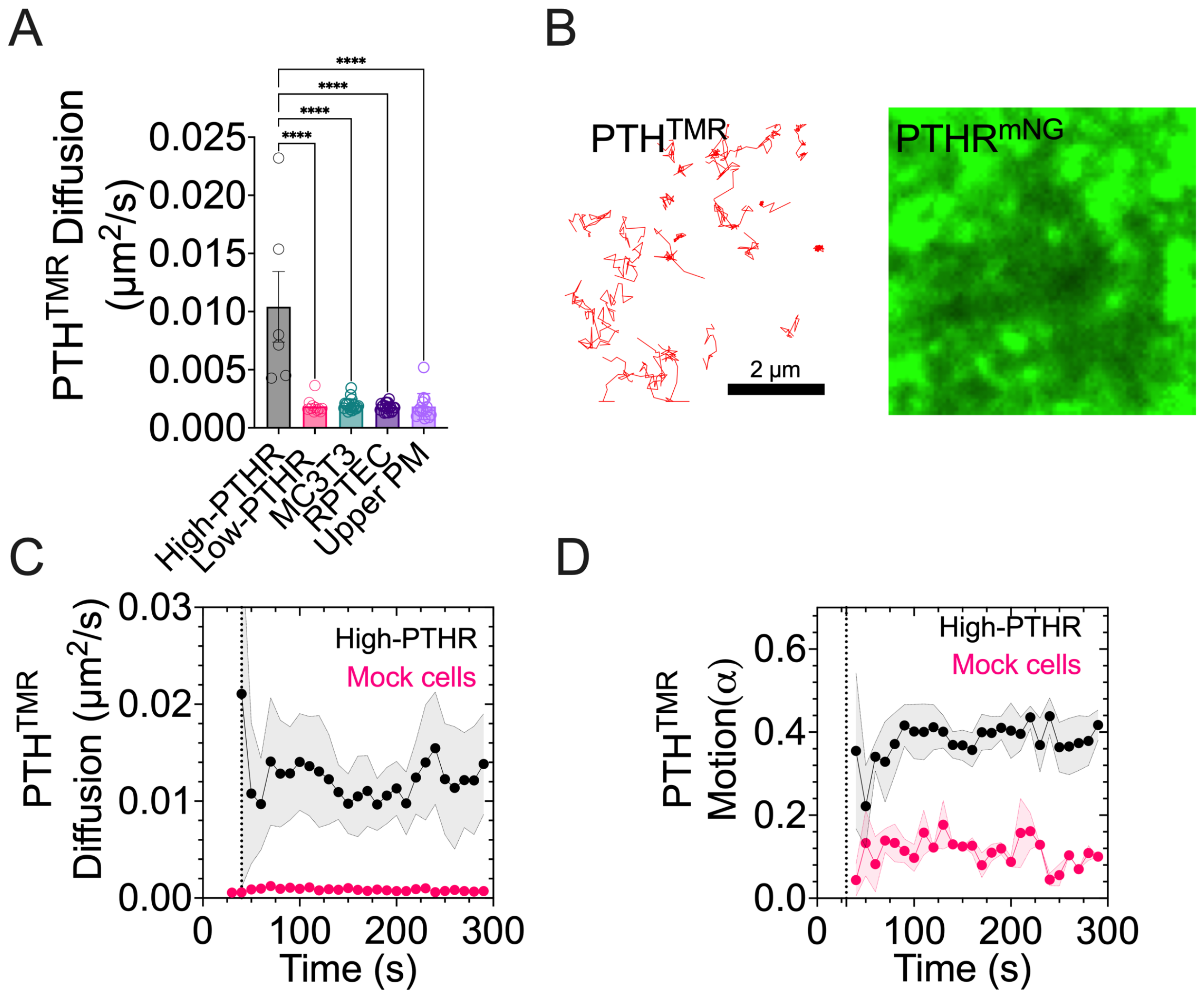

Supplementary Fig. 8| Overexpression of PTH_1R increases the diffusion of $PTHR^{TMR}$. (**A**) Diffusion coefficient of PTH^{TMR} in HEK293 cells with high and low expression of $PTHR^{mNG}$**,** and in cells lines with endogenous expression of PTHR (MC3T3 and RPTEC cells). Similar values were also obtained on the top membrane of HEK293 cells with low expression of PTHR. For comparison, the data presented in low-PTHR, MC3T3, RPTEC, Upper PM are the same as those shown in the panels of figures 2C and 2D (**B**). Example of trajectories of PTH^{TMR} in a cell with high expression of $PTHR^{mNG}$. (**C, D**) Diffusion (C) and motion (D) time-lapses of PTH^{TMR} in cells overexpressing PTHR (black) and in mock cells (magenta). Individual trajectories were analyzed at intervals of 10 seconds. Ligands were added at T = 30 s (vertical dotted line). Data are the mean ± SEM of n = 3 experiments with 2028 trajectories. ****$P < 0.0001$ by one-way ANOVA.

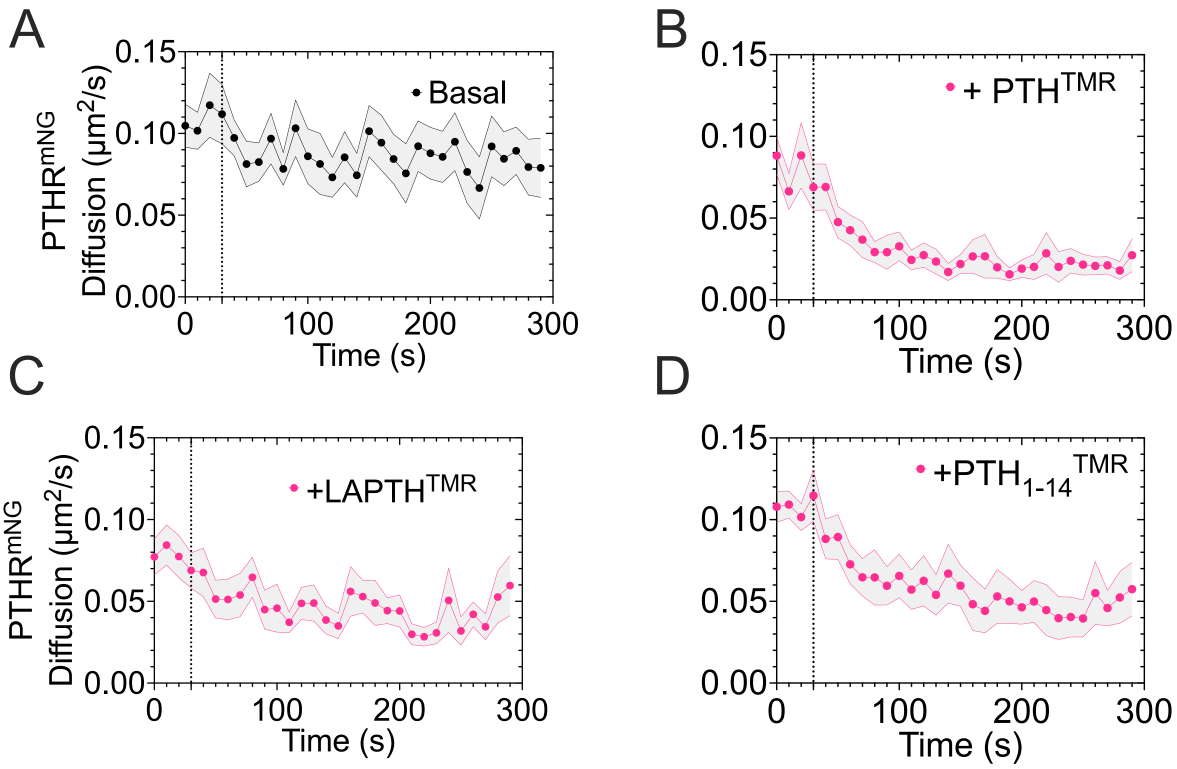

Supplementary Fig. 9| PTH$_1$R diffusion with full agonists. (**A–D**) Diffusion time-lapses of PTH$_1$R^{mNG} alone (A) or in the presence of PTH^{TMR} (B), LA-PTH^{TMR} (C), and PTH$_{1-14}$^{TMR} (D). Data are the mean ± SEM of *n* = 9 (Basal), *n* = 12 (PTH^{TMR}), *n* = 11 (PTH$_{1-14}$^{TMR}), and *n* = 8 (LAPTH^{TMR}) cells for at least 3 independent experiments.

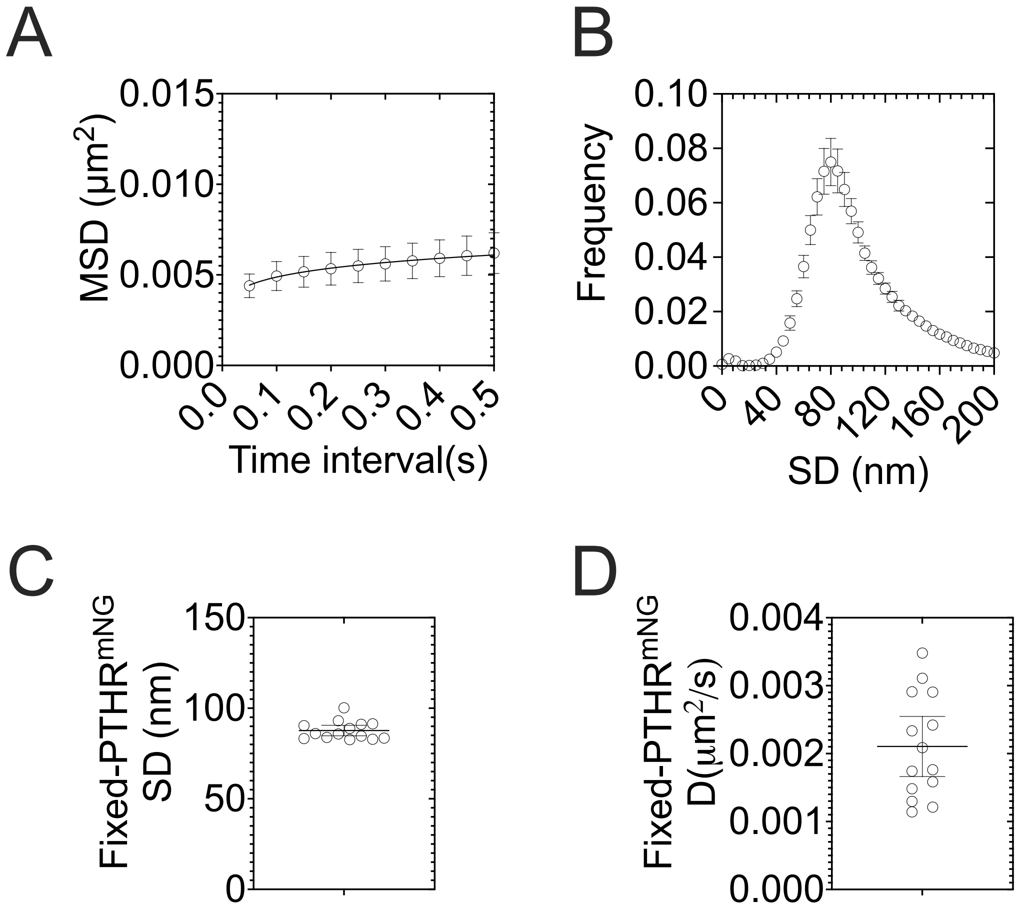

Supplementary Fig. 10| Precision of measurements.
(A) Mean square displacement (MSD) *vs* time intervals of fixed cells expressing PTH_1R^{mNG}. The size of confinement corresponding to this curve was 0.079 µm. Data are the mean ± 95% C.I. of *n* = 14 cells.
(B, C) Frequency distribution of the standard deviation (SD) of the diameter of single molecules. Individual distributions were fit with a gaussian function to extract the mean (C)**.** Data are the mean ± SD of *n* = 14 cells, averaging 46,800 detections/cell.
(D) Lateral diffusion coefficient calculated from data in panel A using the equation MSD = 4Dt = d^2 is ~0.089 µm.

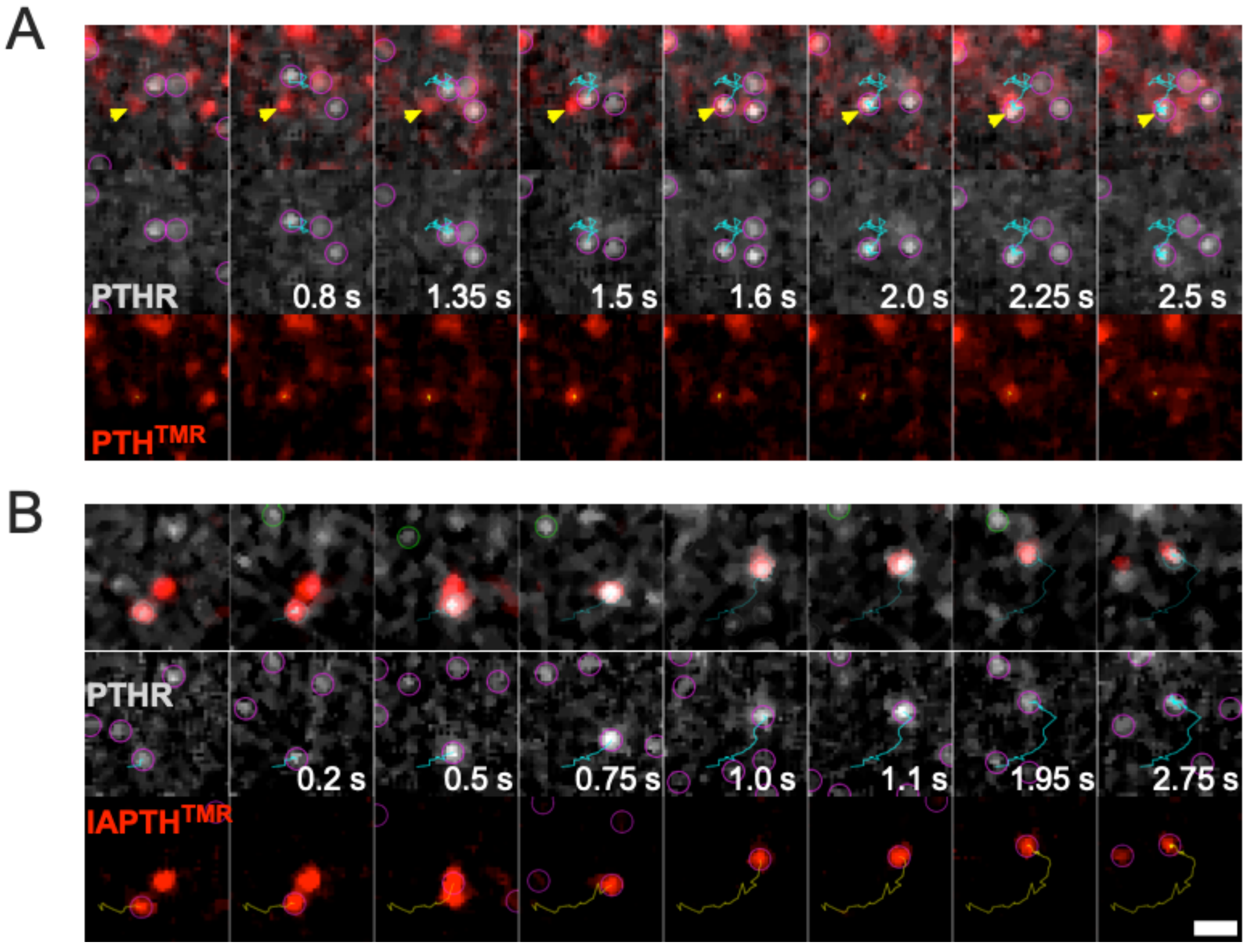

Supplementary Fig. 11| Dual-color single molecule recordings of PTH_1R and fluorescent ligands. (**A**) Representative examples of coincidental detection by single molecule. The PTH_1R diffuses until its collision with a confined PTH^{TMR} molecule (*yellow* arrow). (**B**) The receptor co-diffuses with the antagonist IA-PTH^{TMR}. Scale bar: 1 µm.

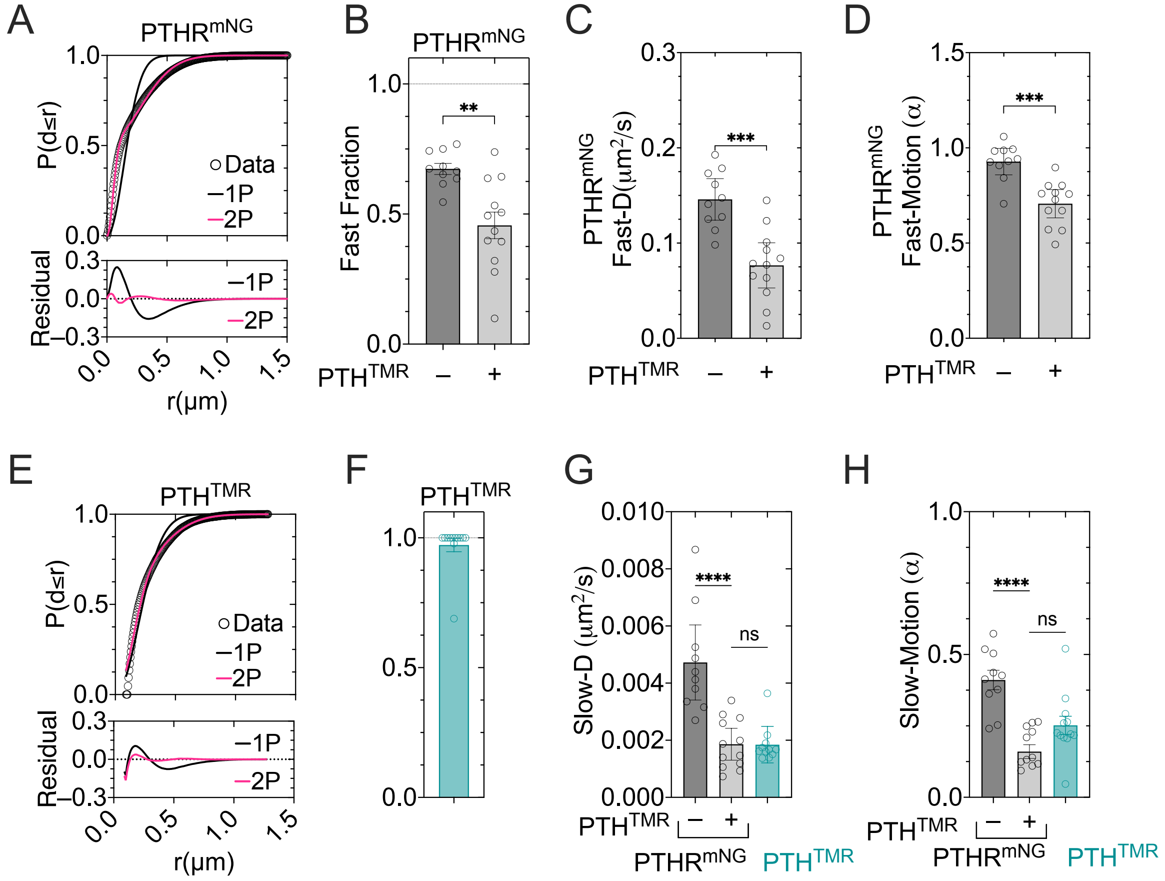

Supplementary Fig. 12| Radial displacement analysis.

(A) Comparing fits of one-population (black line) and two-population (magenta line) models for cumulative distribution of radial displacements at time intervals of 200 ms of PTH_1R^{mNG}. The two-population model accurately describes the experimental data (open circle) for PTH_1R^{mNG}. The corresponding residual plots (i.e., differences between the experimental data and calculated fitted curves) indicate that a two-component model is a better fit.

(B) Fraction of radial displacements corresponding to the fast-diffusing population in the presence or absence of PTH^{TMR} (B). Upon the addition of PTH^{TMR}, there is a significant decrease in the fast fraction, reducing it from 67% to 45%.

(C, D) Diffusion coefficient (C) and motion (D) of the fast-diffusing population in the presence or absence of PTH^{TMR}.

(E) Comparison of diffusion coefficient calculated through MSD for PTH^{TMR} and diffusion coefficient (G) and motion (H) of the slow-diffusing population for PTH_1R^{mNG} in the presence or absence of PTH^{TMR}.

(F) Fraction of radial displacements of PTH^{TMR} The fast-diffusing population for PTH^{TMR} is bigger than 95% indicating a homogeneous diffusion for the ligand.

(G, H) Comparison of diffusion coefficient calculated through MSD for PTH^{TMR} and diffusion coefficient (G) and motion (H) of the slow-diffusing population for PTH_1R^{mNG} in the presence or absence of PTH^{TMR}. Data are the mean ± 95% C.I. of n = 10/179 (PTH_1R^{mNG}), n = 12/70 (PTH_1R^{mNG} + PTH^{TMR}) and n = 12/5786 (PTH^{TMR}) (cells/average trajectories per cell of N = 3 experiments. ****$P < 0.0001$, ***$P < 0.001$, **$P < 0.01$, *$P < 0.05$ by unpaired t-test in panels B, C and D and by one-way ANOVA with a Dunnett multiple comparison in panel G and H.

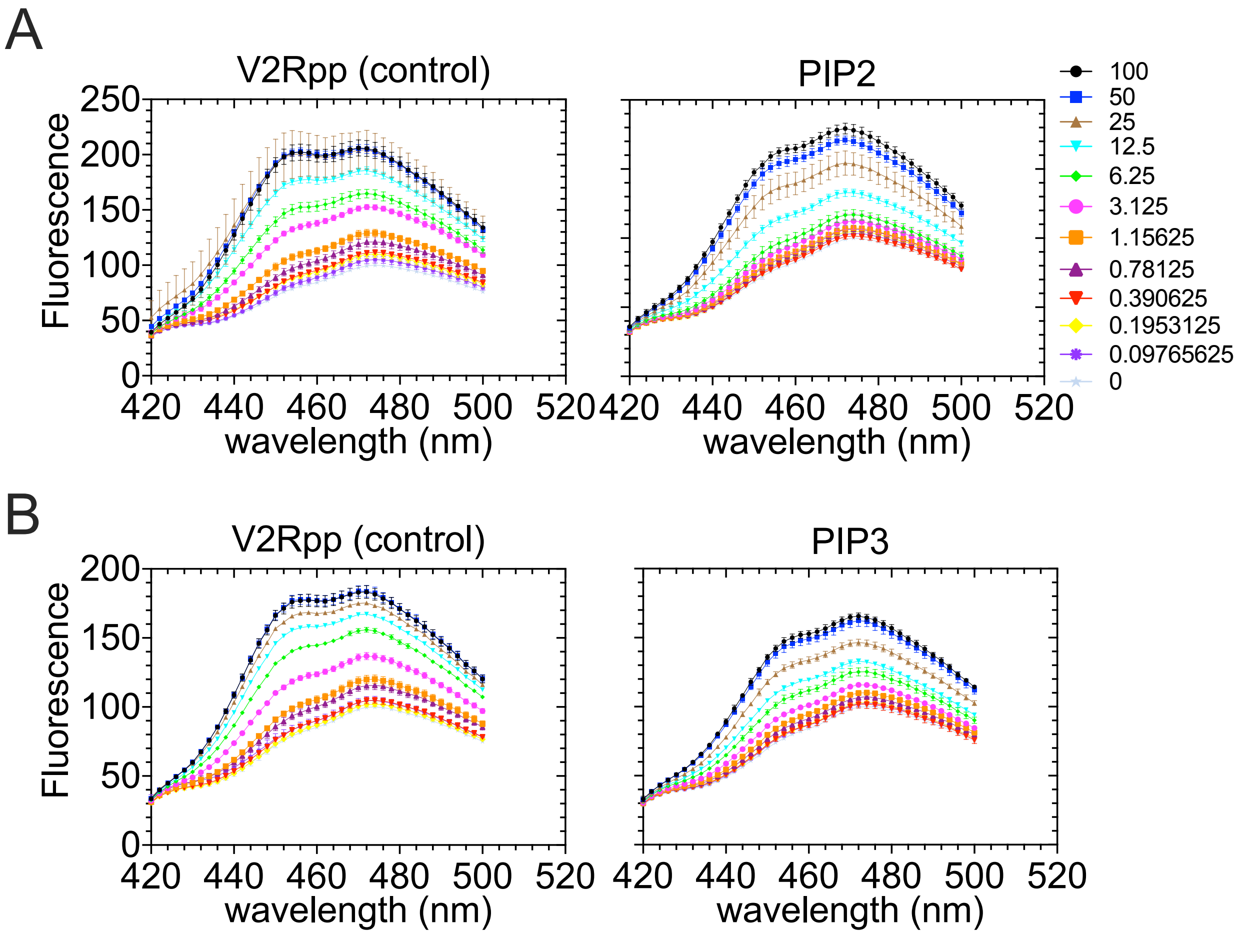

Supplementary Fig. 13| Fluorescence emission spectra of L69C-BIM labeled βarr2 in response to PIP_3. Points are mean ± SD of $n = 3$ experiments and vertical axis corresponds to normalized fluorescence intensity (with 0 μM PIP_2, PIP_3, or V2Rpp as 100).

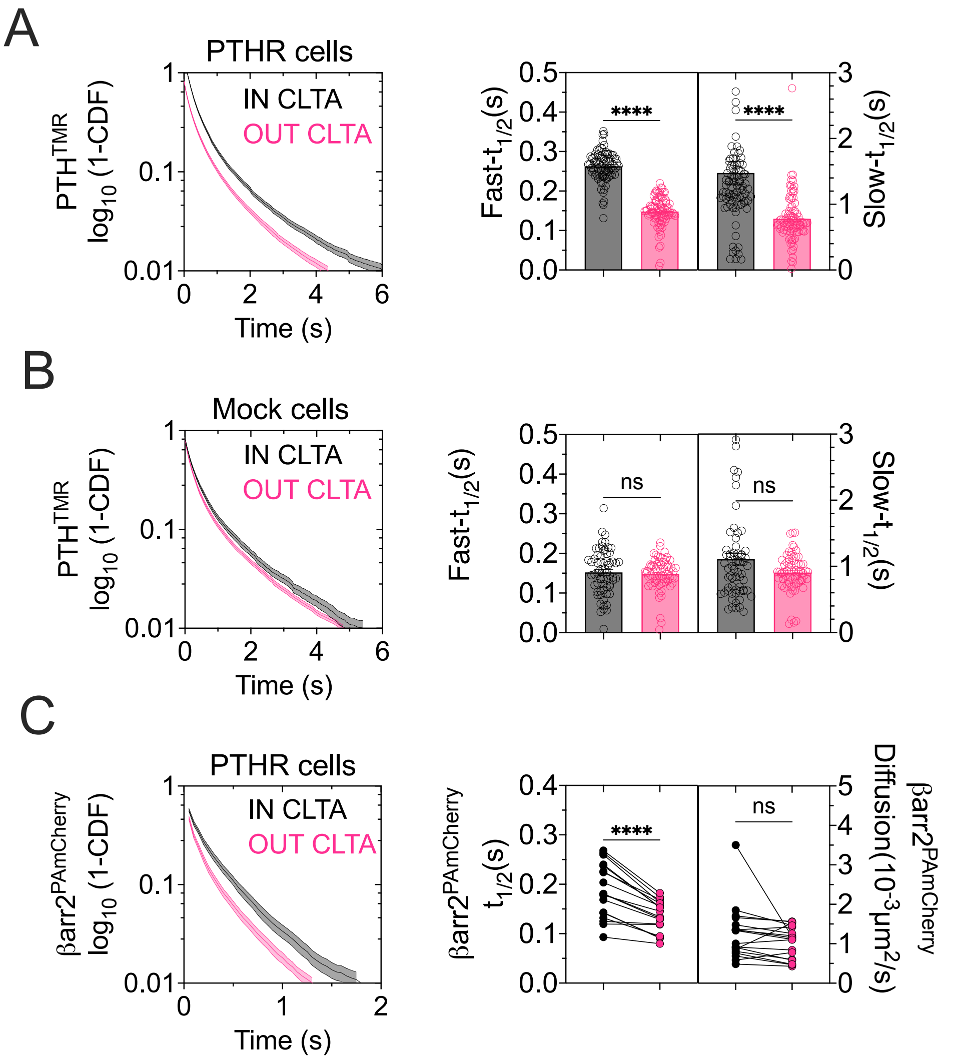

Supplementary Fig. 14| Dwell-time of PTH^{TMR} on clathrin-coated pits.

(**A**, **B**) Survival functions (1-CDF) for the duration of PTH^{TMR} trajectories in endogenously labeled CLTA^{mNG} cells with (A) and without (B) PTH$_1$R expression. Trajectories were categorized based on their location inside (CTLA-IN) or outside (CLTA-OUT) clathrin-coated pits. The survival functions (1-CDF) were fitted using a bi-exponential model to extract the half-life time ($t_{1/2}$) for the fast and slow components. To ensure accurate measurements and exclude newly appearing or disappearing clathrin domains, each time-lapse sequence was divided into 1-minute intervals. This allowed us to generate a binary mask of clathrin domains, providing a precise representation of the existing domains throughout the recording period. Data are the mean ± SEM of *n* = 9 (PTH$_1$R) and *n* = 7 (mock) cells from 3 independent experiments. Data derived from 5 min time-lapse recordings.

(**C**) Survival functions (1-CDF) for the duration of βarr2^{PAmCherry} trajectories in endogenously labeled CLTA^{mNG} cells expressing the PTH$_1$R. The duration of trajectories was count and sorted based on the location inside or outside clathrin-coated pits (left). The survival functions were fit with a single exponential decay to extract the half-life time (center). The diffusion coefficient for the same sorted trajectories were quantified (right). Data are the mean ± SEM of *n* = 18 cells ****$P < 0.0001$, ns, not significant by paired t-test.
