# Supplementary material for: Fast-diffusing receptor collisions with slow-diffusing peptide ligand assemble the ternary parathyroid hormone–GPCR–arrestin complex
Source: Nat Commun. 2024 Dec 3;15:10499. doi: 10.1038/s41467-024-54772-3 (PMC11615292; doi:10.1038/s41467-024-54772-3)
Supplement: Supplementary file 2 — Description of Additional Supplementary Information [file 41467_2024_54772_MOESM2_ESM.docx]

**Description of Additional Supplementary Files**

File Name: Supplementary Movie 1

Description: PTH1R and PTHTMR dualcolor single molecule. A HEK293 cell expressing the PTH1RmNG was stimulated with 10nM of PTHTMR. The region of interest is showed to indicate the area where the analysis is performed to reduce the unspecific binding of PTHTMR to the cover glass. 5 min recording.

File Name: Supplementary Movie 2

Description: PTH1R and PTHTMR coincidental detection. PTH1R (shown in gray) diffuses freely until its collision with immobile PTHTMR molecules (shown in red), which displayed confined motion. For ease of visualization, only the trajectory of the receptor is depicted. The cyan color represents the segments of the trajectory where the receptor is unbound, while the yellow color indicates the segments where colocalization with the ligand is observed (< 80 nm).

File Name: Supplementary Movie 3

Description: PTH1R and IA-PTHTMR coincidental detection. PTH1R (shown in gray) diffuses simultaneously with IA-PTHTMR molecules (shown in red). The yellow trajectory indicates the path of receptor showing coincidental colocalization with the ligand.
